# Changes in e-scooter related maxillofacial injuries following legislative measures in Helsinki, Finland

**DOI:** 10.1038/s41598-025-94602-0

**Published:** 2025-03-21

**Authors:** Johanna Snäll, Henri Vasara, Olli-Jussi Murros, Veli-Pekka Harjola, Maaret Castrén, Tero Puolakkainen

**Affiliations:** 1 Department of Oral and Maxillofacial Diseases, University of Helsinki, P.O. Box 100, 00290 Helsinki, Finland; 2 Helsinki University Hospital, Helsinki, Finland; 3 Department of Emergency Medicine and Services, Helsinki University Hospital and University of Helsinki, Helsinki, Finland

**Keywords:** e-scooter, Trauma, Maxillofacial, Public health, Disease prevention, Dentistry, Dental trauma, Public health

## Abstract

The increasing use of e-scooters globally has resulted in a rise in traffic-related injuries, particularly maxillofacial trauma. This study assesses the potential impact of legislative measures, specifically speed limits and night-time usage restrictions, on reducing maxillofacial injuries from e-scooter accidents. A retrospective cohort study was conducted using data from patients treated for e-scooter-related injuries at Helsinki University Hospital between January 2021 and December 2023. The study included 1275 patients, aged ≥ 16 years, treated in three trauma hospitals. Patients not riding e-scooters at the time of injury were excluded. Injury patterns and the influence of legislative measures were analyzed. Among the 1275 patients, 169 (13.3%) sustained maxillofacial injuries. Legislative restrictions were associated with a significant reduction in maxillofacial injuries, with up to an 88% decrease in some months. Predictors of maxillofacial injuries included older age (OR 1.06, 95% CI 1.04–1.08), alcohol intoxication (OR 3.2, 95% CI 1.5–5.8), and concurrent head and neck injuries (OR 12.1, 95% CI 5.8–25.2). Legislative restrictions on e-scooter use, including speed limits and nighttime riding bans, were associated with a significant reduction in maxillofacial injuries. These findings highlight the importance of targeted policies to mitigate injury risks associated with e-scooter use.

## Introduction

The increasing popularity of standing electronic scooter (e-scooter) usage has had a paramount impact on light traffic from a global perspective. While this mode of transport provides convenient means of traveling and commuting, hazardous and reckless behavior related to e-scooter use has become a global pandemic, and now represents a significant proportion of all traffic-related injuries especially in western countries. Therefore, e-scooter related injuries have emerged as a significant focus of interest from both a clinical and academic perspective.

As the incidence rates of these injuries have increased notably in recent years, specific injury profiles and distinct risk factors related to these accidents have also been recognized. We and others have reported the risks of facial injuries associated with this new type of traffic injury mechanism^1–7^. Previously identified factors associated with e-scooter related facial injuries include riding without a helmet, alcohol use, and night-time riding. In these e-scooter related accidents, facial injuries significantly increase the risk of other injuries especially severe head injuries^1–3^. These widespread and concordant observations have received considerable awareness and concern, which have directly translated into development of preventive measures and restrictions to reduce the amount of sustained e-scooter accidents in general.

Hazardous e-scooter use and related trauma have been considered as sufficiently serious that legislative measures have been put forward. Some major cities have even held referendums that have resulted in the banning of e-scooter rental activity, with the aim of significantly reducing the occurrence rates of these accidents. Lighter measures, including restricting rental e-scooter usage to daytime hours only, have also been implemented. For example, the city of Helsinki set regulations that limited the top speed of e-scooters to 20 km/h during daytime and 15 km/h during night hours as well as prohibited shared e-scooter use during Friday and Saturday nights between 12 and 5 AM. This led to a significant decrease in total e-scooter related injuries^8^. However, there are no reports available on how these restrictions may affect injury rates especially in patients sustaining facial injuries.

This study evaluated specific features of maxillofacial injuries in e-scooter accidents with a particular emphasis on the potential effects of usage restrictions on injury incidence rates. The study hypothesis was that e-scooter related maxillofacial injuries can be reduced with targeted measures.

## Methods

### Study design

This retrospective study at the Helsinki University Hospital included all patients with an injury related to e-scooter riding and presenting to a specific Helsinki emergency department between January 1, 2021 to December 31, 2023.The data were retrieved from the collective electric patient medical records system from three trauma hospitals representing all public hospitals treating patients with acute trauma in Helsinki, Finland (2 level I trauma centers and 1 level IV trauma center.)

Patients were identified from the Helsinki University Hospital data pool using a specific search term from the emergency department patient records. Six e-scooter–related terms with their inflected were used to identify the patients.

### Inclusion and exclusion criteria

All patients aged ≥ 16 years riding an e-scooter in the event of an injury and with comprehensive data on demographic and injury-related variables were included in the study. Patients who sustained their injuries while not riding an e-scooter (for example, pedestrians struck by an e-scooter) were excluded.

### Study variables

The main outcome variable was occurrence of maxillofacial injury, which included any type of hard tissue injury (i.e. facial fractures and dental injuries). Facial fractures were classified based on facial thirds (lower third consisting of mandibular fractures, middle third consisting of different combinations of mid-facial fractures including maxillary, zygomatic, orbital and Le-Fort 1–3 fractures; and upper third consisting of fractures extending to the frontal sinus and upper orbital rim) and different combinations affecting multiple thirds of the facial skeleton. Dental injuries were defined as hard tissue (injuries to the tooth itself) and connective tissue (injuries to the surrounding bone and soft tissues). Soft tissue lacerations were not classified as maxillofacial injuries due to concerns regarding reproducibility and report inconsistencies.

The primary predictor variable was alcohol intoxication, which was confirmed with breathalyzer, blood test, or based on information provided by the patient or the paramedics either on-site or at the emergency department.

Other predictor variables were age, sex and injury-related variables.

Injury-related variables included specific injury mechanism, helmet use, presence of other injuries, subtype of other injuries, and need for hospitalization.

Injury mechanisms were divided into the following three groups: falling over, collision with stationary object (including pedestrians) and collision with a moving vehicle (including automobiles, bicycles and other e-scooters). Other injuries were defined as notable injuries outside the facial skeleton and were categorized and defined as follows: head and neck injuries (defined as traumatic brain injuries, intracranial hemorrhage, blunt cerebrovascular injuries and cervical spine injuries), injuries to the extremities and injuries to the torso (including thoracic, abdominal and pelvic injuries). Time of the injury was categorized as daytime (between hours 0600–1800) or night-time (between hours 1800 − 0600).

In addition, we compared injury occurrence of maxillofacial injuries before and after legislative restrictions. On September 3, 2021, legal measures restricting rental e-scooter use during weekend nights and lowering top speeds came into effect. Information concerning e-scooter availability, restrictions, and top speeds have been reported previously in detail^8^.

### Ethics approval

The study was approved by Helsinki University Hospital research board. According to Finnish legislation on medical research, using public and published data, registry and documentary data, and archive data do not require ethical board processing. Due to the retrospective nature of the study, the Helsinki University Hospital research board waived the need of obtaining informed consent All methods were carried out in accordance with the Declaration of Helsinki.

### Statistical methods

Nominal values are presented as counts and percentages. Age as a continuous variable complied with a Gaussian distribution and therefore was reported using mean and standard deviation (SD).

We examined predictors for maxillofacial injuries with binary logistic regression using the forward stepwise procedure. First, we performed univariable analysis for all potential predictor variables. All injury and patient-related variables with *p* < 0.20 were tested at step zero. The variables with *p* values < 0.05 were left in the final model. The final multivariable model included four variables. We did not detect any considerable multicollinearity between the four variables (tolerance 0.87–0.99). Results from logistic regression are presented as odds ratios (ORs) with 95% confidence intervals (CI). We compared the statistical significance of the patients with and without maxillofacial injuries using the Chi-Square test for nominal values and Student’s *t*-test for age. Statistical analyses were conducted with SPSS version 29.0.0 (IBM corp. release). The level of statistical significance was set as 5% and all tests were two-tailed where appropriate.

## Results

A total of 1275 patients were included in the study of which 169 (13.3%) sustained any type of maxillofacial hard tissue injury (Table 1). The mean ± SD age of patients was 30.4 ± 11.4 years. Males (*n* = 114, 67.5%), alcohol use (*n* = 105, 62.1%) and lack of helmet use (*n* = 157, 92.9%) were all overrepresented in the patients with maxillofacial injuries. Moreover, head and neck injuries occurred in only 5.3% of patients without maxillofacial injuries, whereas the corresponding value was 32.5% in patients presenting with maxillofacial injuries. Most of the injuries occurred during night-time hours in both groups (maxillofacial injury present-group 59.8%, maxillofacial injury absent-group 55.2%.)

**Table 1:** Differences in demographics and clinically relevant variables between maxillofacial and non-maxillofacial injury patients.

Variable	Maxillofacial injury present			Maxillofacial injury absent			*p* value
	No. of patients	%	% of n	No. of patients	%	% of n	
All	169	13.3		1106	86.7		
Age							
Mean (S.D.)	34.2 (12.7)			29.8 (11.1)			< 0.001
Sex							
Male	114	14.3	67.5	683	85.7	61.8	0.15
Alcohol							
Yes	105	24.4	62.1	325	75.6	29.4	< 0.001
Mechanism							
Falling over	146	13.5	86.4	932	86.5	84.3	0.21
Collision with stationary object	18	14.8	10.7	104	85.2	9.4	
Collision with moving vehicle	5	6.7	3.0	70	92.3	6.3	
Helmet use							
Yes	12	26.7	7.1	33	73.3	3.0	0.07
No	81	16.1	47.9	421	83.9	38.1	
No information available	76	10.4	45.0	652	89.6	59.0	
Other injuries							
Yes	72	8.6	42.6	768	91.4	69.4	< 0.001
Type of other injury							
Head and neck	55	48.2	32.5	59	51.8	5.3	< 0.001
Torso	7	10.0	4.1	63	90.0	5.7	0.41
Extremity	26	3.5	15.4	711	96.5	64.3	< 0.001
Time of injury							
Daytime	68	12.3	40.2	495	87.7	43.9	0.27
Night-time	101	14.2	59.8	611	85.8	55.2	

Of the 169 patients with maxillofacial injuries, 121 patients had facial fractures (Table 2). Fractures of the middle third of the facial skeleton were the most frequent fracture type (*n* = 75, 62.0% of facial fracture patients) followed by fractures of the lower third (*n* = 27, 22.3%). Dental injuries were also common as they were seen in 35.5% of all included patients with maxillofacial injuries. Most of the dental injuries were confined solely to hard tissues (*n* = 44, 73.3%).

**Table 2:** Descriptive data of maxillofacial injury profiles sustained by e-scooter riders.

Variable	No.	Total patients	% of total patients		
Maxillofacial injury (any)	169	1275	13.3		

Variable	No.	Total patients	% of total patients	% of facial fractures	% of maxillofacial injuries
Facial fracture (any)	121		9.5	100.0	71.6
Facial fracture type					
Lower third	27		2.1	22.3	16.0
Middle third	75		5.9	62.0	44.4
Upper third	5		0.4	4.1	3.0
Combination of different thirds	14		1.1	11.6	8.3

Variable	No.	Total patients	% of total patients	% of dental injuries	% of maxillofacial injuries
Dental injury (any)	60		4.7	100.0	35.5
Dental injury type					
Hard tissue	44		3.5	73.3	26.0
Soft tissue	12		0.9	20.0	7.1
Both	4		0.3	6.7	3.4

Based on multivariable logistic regression analyses (Table 3), higher age (adjusted OR 1.06, 95% CI 1.04, 1.08, *p* < 0.001), alcohol intoxication (OR 3.2, 95% CI 1.5, 5.8, *p* < 0.001), and other injuries in the head and neck area (OR 12.1, 95% CI 5.8, 25.2, *p* < 0.001) were independent predictors of maxillofacial injuries in patients with e-scooter injuries. On the other hand, patients with extremity injuries had an independent inverse association with maxillofacial injuries (OR 0.15, 95% CI 0.08, 0.29, *p* < 0.001).

**Table 3:** Logistic regression analysis for e-scooter related injuries.

Variable	Univariable			Multivariable		
	Odds ratio	95% confidence intervals	*p* value	Adjusted odds ratio	95% confidence intervals	*p* value
Age	1.03	1.02–1.04	< 0.001	1.06	1.04–1.08	< 0.001
Sex (ref. Male)	1.3	0.9–1.8	0.15			
Alcohol intoxication	3.9	2.8–5.5	< 0.001	3.2	1.8–5.8	< 0.001
Mechanism of injury						
Falling	Ref					
Collision with stationary object	1.1	0.7–1.9	0.7			
Collision with moving vehicle	0.5	0.2–1.1	0.1			
Other injuries (any)	0.3	0.2–0.5	< 0.001			
Type of other injury						
Head and neck	8.6	5.7–13.0	< 0.001	12.1	5.8–25.2	< 0.001
Extremity	0.1	0.07–0.16	< 0.001	0.15	0.08–0.29	< 0.001
Torso	0.7	0.3–1.6	0.41			
Helmet usage*	1.9	0.9–3.8	0.08			
Night-time riding	1.2	0.9–1.7	0.27			

*There were 728 cases where helmet usage was not reported (missing values).

**Fig. 1:**
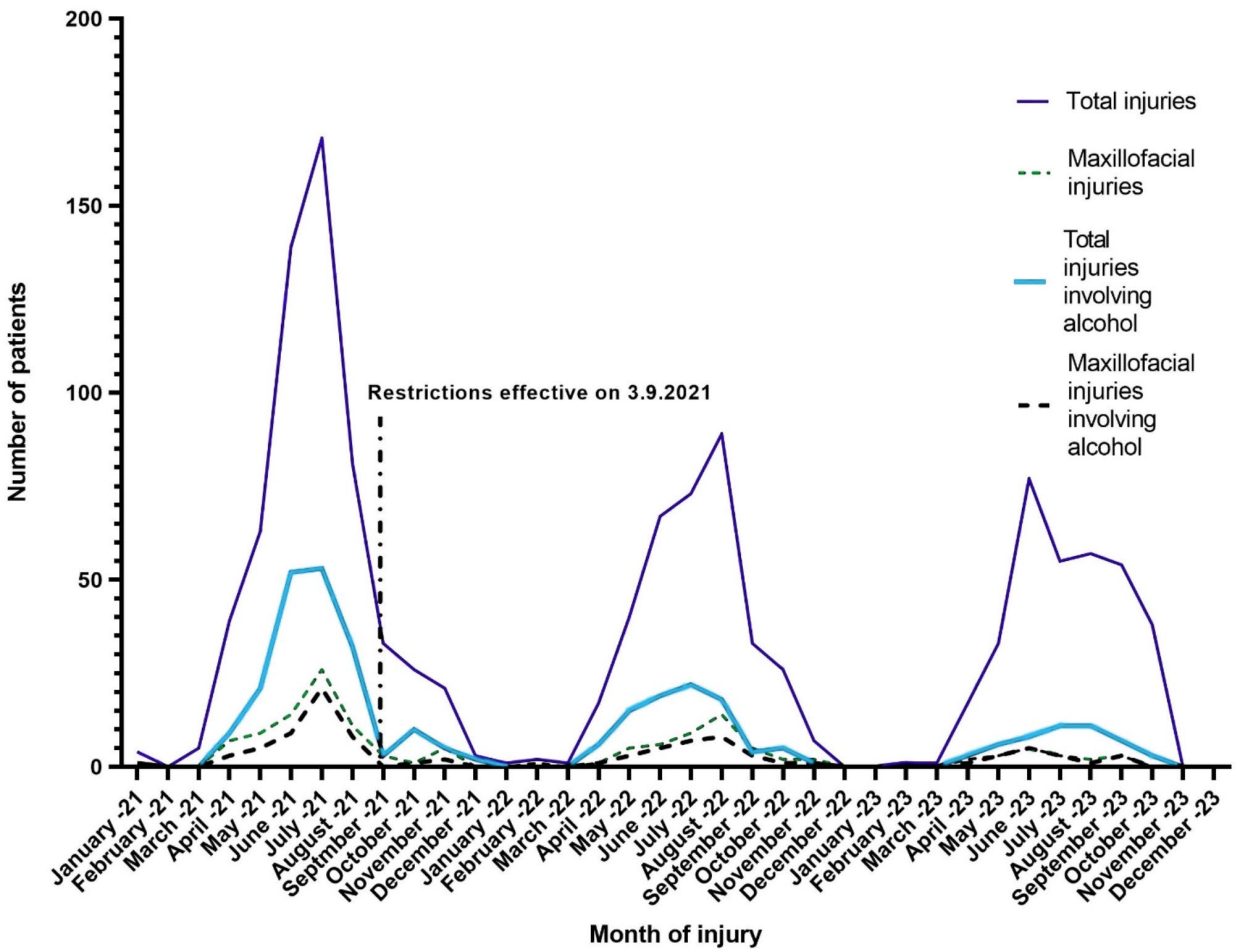
Rates of e-scooter accidents before and after restrictions came into effect. Dark blue, straight lines indicate the numbers of total e-scooter injuries during the study months. Bolded, light blue lines express how many of these accidents took place under the influence of alcohol. Green dashed-lines show the number of maxillofacial injuries sustained. Bolded, dashed-lines present the number of how many of these injuries occurred under the influence of alcohol. Vertical dashed line indicates the date that legislative restrictions came into effect.

When comparing the number of trauma events between corresponding months before and after the restrictions came into force, a salient decrease in e-scooter related maxillofacial injuries can be seen especially during the summer months (Fig. 1). For example, maxillofacial injuries decreased by 57% in June 2022 and by 64% in June 2023 when compared with June 2021. Similarly, maxillofacial injuries were reduced by 65% in July 2022 and by 88% in July 2023 when compared to July 2021. Additionally, a strong relationship between sustained maxillofacial injuries and alcohol use can be observed.

## Discussion

This study provided novel and relevant information on the current phenomena of e-scooter riding with a focus on maxillofacial injuries. In total, 13.3% of included patients sustained a maxillofacial injury. These patients often present with other injuries as well (42.6%), especially concomitant head and neck injuries. Additionally, the majority of patients sustaining maxillofacial injuries where under the influence of alcohol. Importantly, our findings suggest that restrictions in e-scooter usage and speed limits may have contributed to a decrease in the amount of e-scooter injuries where maxillofacial injuries were present.

Previously reported injury rates on e-scooter related facial injuries varied from 23 to 43%^9–11^. More specifically, rates for facial bone fractures varied from 5.3 to 26.1%^9,10,12^. These results are consistent with this study, as our results suggest that almost 10% of all e-scooter related injuries involve facial fractures. Additionally, the frequency of dental injuries in all patients was noteworthy (4.7%). Our results also highlight the frequency of concomitant head and neck injuries sustained by patients with maxillofacial injuries.

Our results suggest that these restrictions may have contributed to a reduction in these injuries as maxillofacial injury rates decreased up to 88% during the summer months when these injuries are most likely to occur. Lower velocities may reduce injury severity as reaction times are increased and transmission of forces may be weakened. On the other hand, contradictory results concerning speed restrictions have also been published. A recent report from Lithuania described that reducing the maximum speed of e-scooters to 15 km/h confined in the city centre did not decrease the actual amount of e-scooter related injuries^13^. This is likely to be explained by the simultaneous soaring of e-scooter use, and other riding conditions than speed including tandem riding, riding under the influence of alcohol, and night-time riding. In addition, it seems that partially enforced speed limits might not be effective enough, as reported by Finnish colleagues where night-time restrictions did not result in a substantial decrease in e-scooter injuries^14^. Therefore, locations and areas with similar legislative measures may present with different outcomes. It is well known that measures aimed to prevent e-scooter abuse are difficult to control and regulate. A significant decrease in injury events can also be achieved by focusing restrictions towards rental companies and the availability of e-scooters, for example by revoking the permits of e-scooter companies^15^. However, our results show that less extreme measures may be even more effective if allocated towards several risk factors simultaneously.

Intoxication is a major predisposing factor for e-scooter related craniofacial injuries as shown before^16,17^. Trivedi et al. reported that patients under the influence of alcohol sustained injuries to the head or facial area in 84% of cases, which is similar to our result of 62%^12^. In the present study, patients under the influence of alcohol were over three times more likely to have maxillofacial injuries than those who were sober. This is most likely due to hindered motor skills and impaired reaction times, and upon collision or falling, the rider is not able to break their fall using their hands or feet, resulting in forces being applied towards the craniofacial skeleton. This is supported by our results and the low incidence of extremity injuries in patients with maxillofacial injuries. Thus, riding while intoxicated must be specifically addressed and restricted by traffic regulations as well as legislative measures to reduce e-scooter related maxillofacial injuries. In Finland, although the blood alcohol limit for driving under the influence is 0.5‰, riding while intoxicated is not strictly controlled. Rental companies have developed pre-rental reaction tests to assess the rider’s condition prior to riding, which may reduce the number of high-risk riders. However, the responsibility lies with the rider who should also be the target audience when increasing awareness of safety measures. This is especially true with private e-scooter users as their compliance towards restrictions is even more challenging to control compared to rental e-scooter users. Therefore, further legislative measures may be warranted to further reduce injury rates.

The lack of helmet use in e-scooter related injuries is also a major concern as shown by other studies^6,10,18^. In the present study, only one in ten patients with facial injuries were reported to wear a helmet at the time of injury. Helmets protect riders against both traumatic brain injuries and facial injuries^19^. A law stating mandatory helmet use while using an e-scooter came into effect in Copenhagen, Denmark in 2022. Siebert et al. studied the helmet use by applying a trained algorithm to automatically register e-scooter helmet use in video data^20^. The passing of the law resulted in an additive effect on helmet use among riders, but total numbers regarding helmet use were still low. Thus, other means of promoting helmet use in relation to legislative measures may be more effective. Unfortunately, incomplete documentation of helmet use in our data does not allow us to further postulate their significance in all injury types.

Another interesting approach to reduce e-scooter injuries is the suggestion that e-scooter riders should hold a valid driving license^21^. As e-scooter usage does not require any theoretical understanding of traffic law, the implementation of some type of driving permit or license could be considered. However, Uluk et al.^22^ demonstrated that these factors did not play a significant role in preventing these accidents. Other regulatory means have also been implemented. For example, Latvian e-scooter companies now require age verification of the rider through their applications. In addition, recent amendments state that e-scooters must display a state registration sticker to be allowed for road-use^17^. Raising further awareness, especially towards e-scooter users and rental companies supplying these vehicles, could be emphasized as well.

The main limitation of the study is its retrospective nature. Due to the study design and patient register data, alcohol intoxication may be underreported. Facial soft tissue injuries were not included in the analyses due to their imprecise definition in the original patient records. More detailed information about the injuries in the facial area could be presented with systematic documentation or prospective study setup. Additionally, the incomplete reporting of helmet use of all patients included is notable. Furthermore, we were unable to reliably address other important confounding variables such as tandem riding or weather conditions that could also predispose the riders to injuries. Additionally, traffic safety is composed of multiple, overlapping factors in addition to speed limits and time-specific restrictions, and future studies should attend to them as entities instead of attributing to each variable separately. Also, the observational setup of the study does not allow to us to draw conclusions on the true effects of legislative measures on preventing e-scooter injuries, and these results should be interpreted accordingly.

Though innocuous by nature from a large-scale perspective, e-scooter use and accidents related to this transport mode have surged in recent years. First identified by surgeons and physicians in emergency departments, the perils and pitfalls of e-scooter use have now incrementally reached the awareness of the general population and affected their perceptions on the safety of these vehicles. This has led to the widespread discussion on the need for proper legislation and referendums aiming to promote traffic safety. Our study suggests that limiting speed limits and night-time use may have reduced the occurrence rates of maxillofacial injuries sustained by e-scooter riders. From a facial traumatologist’ perspective, many injuries could be reduced by paying particular attention to riding under influence of alcohol and further investigating the role of safety helmets.

## Data Availability

According to Finnish legislation, individual patient data cannot be transferred to third parties. Further subanalyses or specifications of the data can be provided based on a reasonable request from Henri.vasara@helsinki.fi.

## References

[1] Arbel, S. et al. Maxillofacial injuries sustained by riders of electric-powered bikes and electric-powered scooters. *Int. J. Environ. Res. Public. Health*, **19**(22). (2022). 10.3390/ijerph192215183 PMC9690219 36429918

[2] Bhaskar, B. et al. A Comparison of Maxillofacial and Head Injuries Following Electric Scooter and Bicycle Accidents. *J. Oral Maxillofac. Surg.*, (2024). 10.1016/j.joms.2024.03.011 38583488

[3] Boschetti, C. E. et al. New generation vehicles: the impact of electric scooter trauma on the severity of facial fractures assessed by FISS score. A multicentre study. *Br. J. Oral Maxillofac. Surg.*, (2024). 10.1016/j.bjoms.2024.05.007 39019685

[4] Faraji, F. et al. Electric scooter craniofacial trauma. *Laryngoscope Investig. Otolaryngol.* **5** (3), 390–395 (2020). 32596481 10.1002/lio2.380 PMC7314474

[5] Kowalczewska, J. et al. Characteristics of E-Scooter-Related maxillofacial injuries over 2019-2022-Retrospective study from Poznan, Poland. *J. Clin. Med.*, **12**(11). (2023). 10.3390/jcm12113690 PMC10253345 37297885

[6] Murros, O. et al. Urban drinking and driving: comparison of electric scooter and bicycle related accidents in facial fracture patients. *Med. Oral Patol. Oral Cir. Bucal*. **28** (3), e238–e246 (2023). 36243995 10.4317/medoral.25662 PMC10181023

[7] Thoenissen, P. et al. Patterns of craniomaxillofacial trauma after E-Scooter accidents in Germany. *J. Craniofac. Surg.* **32** (4), 1587–1589 (2021). 33867518 10.1097/SCS.0000000000007694

[8] Pakarinen, O. et al. Speed and nighttime usage restrictions and the incidence of shared electric scooter injuries. *JAMA Netw. Open.* **6** (11), e2341194 (2023). 37921765 10.1001/jamanetworkopen.2023.41194 PMC10625032

[9] Bracher, A. I. et al. Trauma characteristics associated with E-Scooter accidents in Switzerland-A case series study. *Int. J. Environ. Res. Public. Health*, **20**(5). (2023). 10.3390/ijerph20054233 PMC10002011 36901244

[10] Lavoie-Gagne, O. et al. Characterization of electric scooter injuries over 27 months at an urban level 1 trauma center. *Am. J. Emerg. Med.* **45**, 129–136 (2021). 33690079 10.1016/j.ajem.2021.02.019

[11] Sheikh, M. et al. Electric scooter related injuries in Calgary emergency departments. *CJEM* **24** (7), 735–741 (2022). 36287208 10.1007/s43678-022-00378-x

[12] Trivedi, T. K. et al. Injuries associated with standing electric scooter use. *JAMA Netw. Open.* **2** (1), e187381 (2019). 30681711 10.1001/jamanetworkopen.2018.7381 PMC6484536

[13] Suslavicius, K. A. et al. Unveiling the surge: A comprehensive analysis of E-Scooter-Related injuries at an urban level 1 trauma center in Vilnius, Lithuania (2018–2021). *Cureus* **16** (2), e54616 (2024). 38523964 10.7759/cureus.54616 PMC10959149

[14] Liukkonen, R. et al. Association of nighttime speed limits and electric Scooter-Related injuries. *JAMA Netw. Open.* **6** (6), e2320868 (2023). 37382959 10.1001/jamanetworkopen.2023.20868 PMC10311383

[15] Markowitz, M. et al. The impact of the City of Miami’s decision to revoke electric scooter company permits on orthopedic trauma at a level I trauma center. *J. Emerg. Med.* **66** (2), 177–183 (2024). 38290883 10.1016/j.jemermed.2023.10.024

[16] Cevik, J. et al. The impact of electric scooters in Melbourne: data from a major trauma service. *ANZ J. Surg.* **94** (4), 572–579 (2024). 38087881 10.1111/ans.18814

[17] Saulitis, A. et al. Characteristics and injury patterns in traumatic brain injury related to E-Scooter use in Riga, Latvia: Multicenter case series. *Med. (Kaunas)*, **60**(4). (2024). 10.3390/medicina60040540 PMC11051852 38674186

[18] Stray, A. V. et al. Characteristics of electric scooter and bicycle injuries after introduction of electric scooter rentals in Oslo, Norway. *JAMA Netw. Open.* **5** (8), e2226701 (2022). 35969397 10.1001/jamanetworkopen.2022.26701 PMC9379742

[19] Stassen, H. S. et al. Effect of helmet use on maxillofacial injuries due to bicycle and scooter accidents: A systematic literature review and meta-analysis. *Int. J. Oral Maxillofac. Surg.* **53** (1), 28–35 (2024). 37031014 10.1016/j.ijom.2023.01.013

[20] Siebert, F. W. et al. Computer vision-based helmet use registration for e-scooter riders—The impact of the mandatory helmet law in Copenhagen. *J. Saf. Res.* **87**, 257–265 (2023). 10.1016/j.jsr.2023.09.021 38081699

[21] Jones, K. et al. Oral and maxillofacial injuries associated with e-scooter use at broomfield hospital: a cohort study of 24 months of data since e-scooter legalisation in the UK. *Br. Dent. J.*, 1–5. (2023). 10.1038/s41415-023-5506-5 PMC9897602 36737457

[22] Uluk, D. et al. E-scooter incidents in Berlin: an evaluation of risk factors and injury patterns. *Emerg. Med. J.* **39** (4), 295–300 (2022). 34099458 10.1136/emermed-2020-210268 PMC8961771

